# Elucidation of B-cell specific drug immunogenicity liabilities via a novel *ex vivo* assay

**DOI:** 10.3389/fimmu.2025.1589483

**Published:** 2025-06-04

**Authors:** Cary M. Looney, Axel Ducret, Guido Steiner, Karen Dernick, Katharina Hartman, Michel Siegel, Timothy Hickling, Alex Odermatt, Céline Marban-Doran

**Affiliations:** ^1^ Roche Pharmaceutical Research and Early Development, Pharmaceutical Sciences, Roche Innovation Center Basel, Basel, Switzerland; ^2^ Division of Molecular and Systems Toxicology, Department of Pharmaceutical Sciences, University of Basel, Basel, Switzerland

**Keywords:** immunogenicity, biotherapeutics, anti-drug antibodies, *in vitro* B-cell assay, assay development

## Abstract

The advent of large molecule therapeutics has revolutionized treatment options for previously unmet medical needs. This advent has also led to an increased impact of immunogenicity on drug efficacy and safety. In order to maximize the potential of large molecule therapeutics, immunogenicity-related liabilities must be identified as early in development as possible, using an integrated risk assessment that takes into account the various cell types and processes involved. Here, we describe the development of an *ex vivo* B-cell immunogenicity assay, to capture a key component of the immune response that has been missing from previously published *ex vivo* immunogenicity assays. Plasmablasts/plasma cells were preferentially expanded in this assay, a subset of which were drug-specific and presented drug-specific peptides on MHC Class II. This assay represents an important tool in the immunogenicity risk assessment toolkit, to allow liabilities to be identified and mitigated early in the drug development process.

## 1 Introduction

Novel therapies continue to be developed, expanding the options for previously intractable diseases. From engineered antibodies (Ab) to gene therapy, the quest to address unmet medical needs continues to expand its array of innovative solutions. The efficacy and safety of these new therapeutics, however, continues to be restricted due, in part, to the risk of immunogenic responses.

Immunogenicity – an unwanted immune response to an administered drug, characterized by the presence of anti-drug antibodies (ADAs) – is a risk generally seen with large molecule therapeutics, peptides, and gene therapies. It can negatively impact drug efficacy, either via neutralizing antibodies (NAb) that block the binding of the drug to its target, antibody-mediated rapid clearance of the drug, or both. It can also negatively impact safety, as an immune response and the corresponding cytokine release can be harmful, potentially even life-threatening (1).

Therefore, predicting and controlling immunogenicity early on in clinical development is critical for advancing safe and effective drugs through the pipeline; indeed, regulatory bodies (including the European Medicines Agency (EMA) and the Food and Drug Administration (FDA)), are encouraging drug developers to consider immunogenicity risk as early as possible in the development process, to minimize risks to subjects and patients (2, 3).

New tools to evaluate immunogenicity are being increasingly adopted. Recent advancements in *in silico* tools have led to the development of models that estimate the humanness of antibody sequences (4–6). Despite these innovations, most models exhibit weak correlations with ADA rates, underscoring the necessity to further explore not just T cell antigenicity but also antigen uptake, processing and presentation as well as B cell antigenicity using additional in silico and *in vitro* tools. B-cells play a critical role in the immune response. Not only are they the source of ADAs, but as professional antigen-presenting cells (APCs) in turn, they can help coordinate an anti-drug immune response and secrete inflammatory cytokines that are a key part of harmful responses to drugs (7).

To date, published models of immunogenicity are currently far from being able to reliably predict clinical immunogenicity incidences (8–10); indeed, clinical immunogenicity itself can vary greatly depending on the population in question, disease background, concurrent medications, and myriad other factors that influence immunogenicity. *Ex vivo* and preclinical immunogenicity assays are intended to rank the immunogenicity potential of drugs, identify immunogenic epitopes to avoid, inform mitigation strategies to decrease immunogenicity, and better understand the immunogenic response.

In the interest of creating a tool to inform immunogenicity from the B-cell standpoint, a cell type that has not been well-addressed by previously published immunogenicity risk assessment tools, we have developed an *ex vivo* B-cell immunogenicity assay. This assay is based on stimulation of healthy donor peripheral blood mononuclear cells (PBMCs) by IL-2, IL-4, and CpGs, with a subsequent readout of drug-specific B-cells. The ability to identify drug-specific B-cells via specific detection reagents for the stimulating drug allows for comparison of the degree of B-cell stimulating capacity of different drugs, analysis of the effect of the removal of potential immunogenic epitopes, and sorting of specific cells for B-cell receptor (BCR) sequencing and analysis of presented peptides.

Given the complexity of the immune response and the high variability in observed clinical immunogenicity, it is necessary to use many tools in combination and take into account all immunogenicity risk factors for optimal prediction and management of anti-drug immune responses. The B-cell assay reported here is a key part of such a toolkit.

## 2 Materials and methods

### 2.1 PBMC-based B-cell immunogenicity assay

The assay was run on PBMCs taken from anonymized healthy donors. 50 mL of blood was drawn into lithium heparin tubes, and PBMCs were purified using SepMate PBMC isolation tubes (StemCell 85450) with Ficoll (Cytiva 17144003), using manufacturer’s instructions. In brief, 15 mL of Sepmate was added to the tubes, and the tubes were pulse-spun. 50 mL of blood was then slowly layered on top of the Ficoll, and the tubes spun for 10 min at 1200 g. Cells were poured off into fresh 50 cc conical tubs (Falcon 353070), topped up to 50 mL with 1x PBS (Gibco 14040-091), and spun at 300 g for 15 min. The supernatant was aspirated, and cells resuspended in 10 mL of 1x PBS by pipetting. Cells were resuspended in eDRF made of 1:1 RPMI1640 (Gibco, A10491-01):DMEM-F12 (Gibco 31331-028) with 10% fetal bovine serum (FBS) and 0.25 mM L-Leucyl-L-Leucine methyl ester (LLME; Cayman 16008–250 mg) at 1 x 10^7^ cells/mL. Cells were pelleted and washed after 20 min of incubation, then resuspended at 1 x 10^7^ cells/mL in eDRF with 5 μM Class A CpG (ODN 2216; LabForce customized, LABtlrl-2216-1) and 20 ng/mL of IL-2 (R&D Systems, 202-IL-010/CF) and IL-4 (R&D Systems, 6507-IL-010/CF), with either no antigen as a control or 100 μg/mL antigen. The positive control stimulation was keyhole limpet hemocyanin (KLH-Imject Maleimide-Activated mcKLH, Thermo Fisher Scientific, 77600); external drugs were purchased from the local pharmacy, while Roche drugs were taken from internal stock. Drug formulations were as follows: CEA-IL2v, 10 mg/mL; CEA-TCB, 19.2 mg/mL; CEA-IgG, 5.5 mg/mL; Roche Compound 1, 4.8 mg/mL; Roche Compound 1 Parental, 6.2 mg/mL; Roche Compound 2, 6.5 mg/mL; Roche Compound 2 Parental, 3.2 mg/mL, all these compounds were formulated in 20 mM histidine, 140 mM NaCL, pH 6. Natalizumab (100 mg/mL) was provided as a solution in 0.48 mg/mL sodium phosphate, 8.2 mg/mL sodium chloride (NaCl), 0.2 mg/mL polysorbate 80 (p80), pH 6.1. Infliximab (10 mg/mL) was provided in a solution of 50mg/mL sucrose, 0.05 mg/mL p80, and 0.83 sodium phosphate, pH 7.2. Adalimumab (40 mg/mL) was provided in a solution of 6.2 mg/mL sodium chloride, 2.4 mg/mL sodium phosphate, 0.3 mg/mL sodium citrate, 1.3 mg/mL citric acid, 12mg/mL mannitol and 0.5 mg/mL p80, pH 5.2. Basiliximab (5 mg/mL) was provided in a solution of 1.4 mg/mL potassium phosphate, 0.2 mg/mL hydrogen phosphate, 0.32 mg/mL sodium chloride, 4 mg/mL sucrose, 16 mg/mL mannitol, and 8 mg/mL glycine, pH 6. Bevacizumab (25 mg/mL) was provided in a solution of 6mg/mL trehalose dehydrate, 0.58 mg/mL sodium phosphate, and 0.04 mg/mL polysorbate 20, pH 6.2. Pembrolizumab (25 mg/mL) was provided in a solution of 1.55 mg/mL L-histidine, 70 mg/mL sucrose, 0.2 mg/mL p80, pH 5.6. Tocilizumab (20 mg/mL) was provided in a solution of 0.147 mg/mL L-arginine, 20.9 mg/mL L-arginine hydrochloride, 20 mM L-histidine, 20 mM L-histidine hydrochloride monohydrate, 4.5 mg/mL L-methionine, 0.2mg/mL polysorbate 80, pH 6.0. Ustekinumab (5 mg/mL) was provided in a solution of 0.02 mg/mL EDTA, 1.8 mg/mL L-histidine, 0.4 mg/mL L-methionine, 0.4 mg/mL p80, and 85 mg/mL sucrose, pH 5.7. Durvalumab (50 mg/mL) was provided in a solution of 0.2 mg/mL histidine, 10.4 mg/mL trehalose dihydrate, 0.02 mg/mL p80, pH 6.0.

Cultures were restimulated at Day 4 with 5 μM Class B CpG (ODN 2006; Lab-Force customized, LABtlrl-2006-1) and 20 ng/mL of IL-2 and IL-4.

Cells were harvested for flow cytometry analysis at Day 7. For detection of antigen-specific B-cells by directly conjugated antigen, cells were pelleted and washed at Day 6, resuspended in eDRF with 5 μM Class B CpG (ODN 2006; LabForce customized, LAB-tlrl-2006-1), 20 ng/mL of IL-2 and IL-4, and 100 μg/mL labeled stimulating antigen.

Labeled antigens were prepared using an Alexa Fluor 647 Antibody Labeling Kit (Invitrogen, A20186) or Alexa Fluor 680 Antibody Labeling Kit (Invitrogen, A20188), following the manufacturer’s instructions.

### 2.2 Whole blood-based B-cell immunogenicity assay

The whole blood assay was run on blood taken from anonymized healthy donors. 10 mL of blood was drawn into lithium heparin tubes, and 100 μL of blood added to a 96-well plate (ADD) with 5 μM Class A CpG (ODN 2216; LabForce customized, LABtlrl-2216-1) and 20 ng/mL of IL-2 (R&D Systems, 202-IL-010/CF) and IL-4 (R&D Systems, 6507-IL-010/CF), with either no antigen as a control or 100 μg/mL stimulating antigen. Positive control stimulation and labeled drugs were as in 2.1, above.

Cultures were restimulated at Day 4 with 5 μM Class B CpG (ODN 2006; Lab-Force customized, LABtlrl-2006-1) and 20 ng/mL of IL-2 and IL-4. Cells were harvested for flow cytometry analysis at Day 7.

### 2.3 Flow cytometry

Harvested cells were transferred to a 96-well round-bottom plate (Granier Bio-One Cellstar #650180). Cells were pelleted, washed with 100 μL flow buffer (Biolegend, 420201), and resuspended in 50 μL Brilliant Stain Buffer (BD Pharmingen, 566349) with 2.5 μL Fc block (BD Pharmingen, 564220)

Cells were stained with panels containing antibodies to CD3, CD4, CD19, CD20, CD27, CD38, CD45, Ki-67, KLH (BD Pharmingen 563769, 557695, 563325, 560734, 567181, 555459, 564099, 558615, 612758), and an internally generated antibody against the hIgG1-P329G LALA Fc region (9), used in many Roche antibodies, conjugated to Alexa Fluor 647.

For cytokine analysis of T-cells, Brefelden A (BD Pharmingen 555029) was added to wells at a concentration of 1 μL/mL and incubated for 4 hours. Cells were stained extracellularly and then intracellularly as described above, with antibodies against IL-2, IL-4, IL-6, IL-7A and IFNγ (BD Pharmingen 551383, 612835, 559331, 560799, 560742).

Cells were stained at room temperature for 20 min, covered with foil to protect them from light. Cells were washed with PBS and resuspended in Cytofix (BD Pharmingen 554655), then incubated at 4 ^˚^C for 20 min. For intracellular staining, cells were washed with 1x BD Cytoperm (BD Pharmingen 561651), then resuspended in 50 μL 1x Cytoperm and intracellular antibodies, then incubated at 4 ^˚^C for 20 min. Cells were washed and analyzed on a BD Fortessa (BD Biosciences, Franklin Lakes, NJ, USA).

### 2.4 Data analysis

FlowJo 10.8.1 (FlowJo LLC, Ashland, OR, USA) was used to analyze FCS files; data was exported to Tibco Spotfire 12.0.4 for analysis and figure generation (TIBCO Software, Palo Alto, CA, USA).

Data management and statistical analysis were performed in the R programming language (https://www.R-project.org/, version 4.3.0), including essential packages for handling generalized linear models (nlme 3.1-163, emmeans 1.8.7), robust linear models (MASS 7.3-60) and ROC curve analysis (pROC 1.18.4).

Percentage of positive cells were directly calculated from the cell count data. Stimulation Indices (SI) were determined as fold changes of those percentages from the donor matched baseline (NoAg). This is a common procedure to handle donor specific baseline impact but often leaves behind a systematic correlation in the data where donors with a higher baseline show overall lower stimulation indices for the test items. We thus generally also calculate corrected fold changes (SIcorr) using a linear model approach to level out this systematic trend, essentially increasing fold changes for higher pre-stimulated donors based on the baseline value.

For the whole blood immunogenicity assay we tried to obtain a first idea whether the outcome may be to some point predictive of a clinical outcome. Because of the very limited compound set at this stage, which made it impossible to set apart a dedicated test set for any prediction algorithm, we instead determined via grid searching some thresholds for averaged SIcorr and clinical ADA rate (on a compound basis) that led to an optimal separation of low and high immunogenic compounds based on assay results, using the clinical ADA outcome as ground truth. AUC (area under curve) was used as a global measure for class separability. To assess how significant this optimal AUC value is, we repeated this approach many times with random permutations of clinical ADA rate labels, thus providing a null distribution.

### 2.5 Confocal imaging

Total B-cells were isolated from Day 7 cultures using the Easysep B-cell isolation kit (Stemcell, 17954) per manufacturer’s instructions. Cells were stained with antibodies against CD19 and CD79b (BD Pharmingen 557931 and 557697), Hoechst nuclear stain (Invitrogen R37165), and either labeled antigen or anti-PGLALA antibody, as described above. Cells were stained and fixed as described for Flow Cytometry, above, then transferred to an optical plate (Perkin-Elmer 6055802) and pelleted for 3 min at 300 g.

High-content confocal imaging was performed using an Opera Phenix (Perkin Elmer), using a 63× water immersion lens. 340 fields of view (each 39 mm^2^) were imaged for each well at 5% overlap, with 6 z-stacks per field at 2-μm intervals to ensure comprehensive imaging of the B-cell monolayer. Data are representative of three separate experiments, each with four separate donors. Image analysis was performed using Harmony (v4.9). Optical correction was performed using bright-field correction.

### 2.6 MHC-II associated peptide proteomics

B-cells and monocyte-derived dendritic cells (moDCs) were sorted via fluorescence-activated cell sorting (FACS) from Day 7 cultures, gated on FSC/SSC lymphocytes, SSC-A/SSC-H single cells, CD3^neg^CD19^+/lo^ B-cells, and FSC/SSC monocytes, SSC-A/SSC-H single cells, CD3^neg^CD19^neg^CD11c^+^ moDCs. These samples were lysed in protein Lo-Bind tubes (Eppendorf 022431081) in buffer containing 29 mM Tris-HCl pH 7.8, 5 mM MgCl2, 1% Triton X-100, and 1x Pierce protease inhibitor tablet (ThermoScientific, A32955), on a shaker for 1 hour at 4°C. MHC-II peptide-receptor complexes were immunoprecipitated overnight at 4°C using an anti-HLA-DR biotin-conjugated antibody (RayBiotech, 150-10306) on a rotator.

Isolation of the HLA-DR receptors and the subsequent elution of the associated MHC-II peptides was performed using the AssayMAP Bravo platform (Agilent Technologies, 3029078) and accompanying streptavidin cartridges (Agilent Technologies, G5496-60021) as described elsewhere (11).

#### 2.5.1 LC-MS/MS method

Peptide samples were directly loaded onto Evosep C18 tips (Evosep; Cat: #EV2001) according to the manufacturer’s recommendations and stored at 4°C until LC-MS/MS analysis. Peptide samples were analyzed in a trapped ion mobility time-of-flight mass spectrometer (TimsTOF PRO 2, Bruker) equipped with a Captive electrospray source operated at 1200–1400 V. Peptides were separated by reverse-phase chromatography using an Aurora Elite column (15 cm x 75 μm i.d., 1.7 μm particle size, heated at 45 ˚C; Ion Opticks) using an Evosep One standardized nanoLC platform (Evosep; EV-1000). A turnaround time of 31 min was achieved using the Evosep’s built-in program 40 SPD Whisper. Eluted MHC-II peptides were analyzed by data-directed analysis following standard operating parameters. The TIMS accumulation/ramping time was set to 150 ms (mobility range: 0.6-1.6) while the TOF analyzer was set to record ions in the mass range m/z 100-1700 (global cycle time: 1.71 sec). One survey scan (selection range for MS/MS analysis: m/z 350-1300, ion mobility 0.7-1.5, 1<z<6) was followed by MS/MS analysis in PASEF mode including up to 10 TIMS ramps per full cycle. Dynamic exclusion prevented the repeated selection of an ion for MS/MS analysis for 9 sec.

#### 2.5.2 LC-MS/MS data analysis

The LC-MS/MS raw data files were analyzed using the PEAKS Studio software (version XPro, Bioinformatics Solutions Inc.). The data was searched against the human protein database UniProtKB (http://www.uniprot.org, release 2015_10, 88500 TrEMBL and SwissProt entries containing the amino acid sequences of the test therapeutic proteins). Searches were performed with a tolerance of 15.0 ppm (precursor mass) and 0.05 Da (fragment ions) using the unspecific digest mode. Met-sulfoxide, Asn/Gln de-amidation, and N-terminal pyro-glutamylation were considered as dynamic modifications. Peptide results were reported at 1% false discovery rate cutoff, and exported in a tab-delimited table without further normalization. Heat maps and accompanying diagrams were generated with dataMAPPs - an in-house developed R-based workflow for the quality control, processing, and analysis of MAPPs data (12). The binding of identified peptides to HLA-DRB receptors was analyzed using the NetMHCIIpan-4.2 server (13) with recommended settings (14).

#### 2.5.3 Ethics

Ethical approval was not required for the studies involving humans because blood or peripheral blood mononuclear cells (PBMCs) from healthy donors were sourced from the Roche Employee Blood Donation Program in Basel, Switzerland. The studies were conducted in accordance with the local legislation and institutional requirements. The participants provided their written informed consent to participate in this study.

## 3 Results

### 3.1 Development of an *ex vivo* PBMC-based B-cell assay

**Figure 1:**
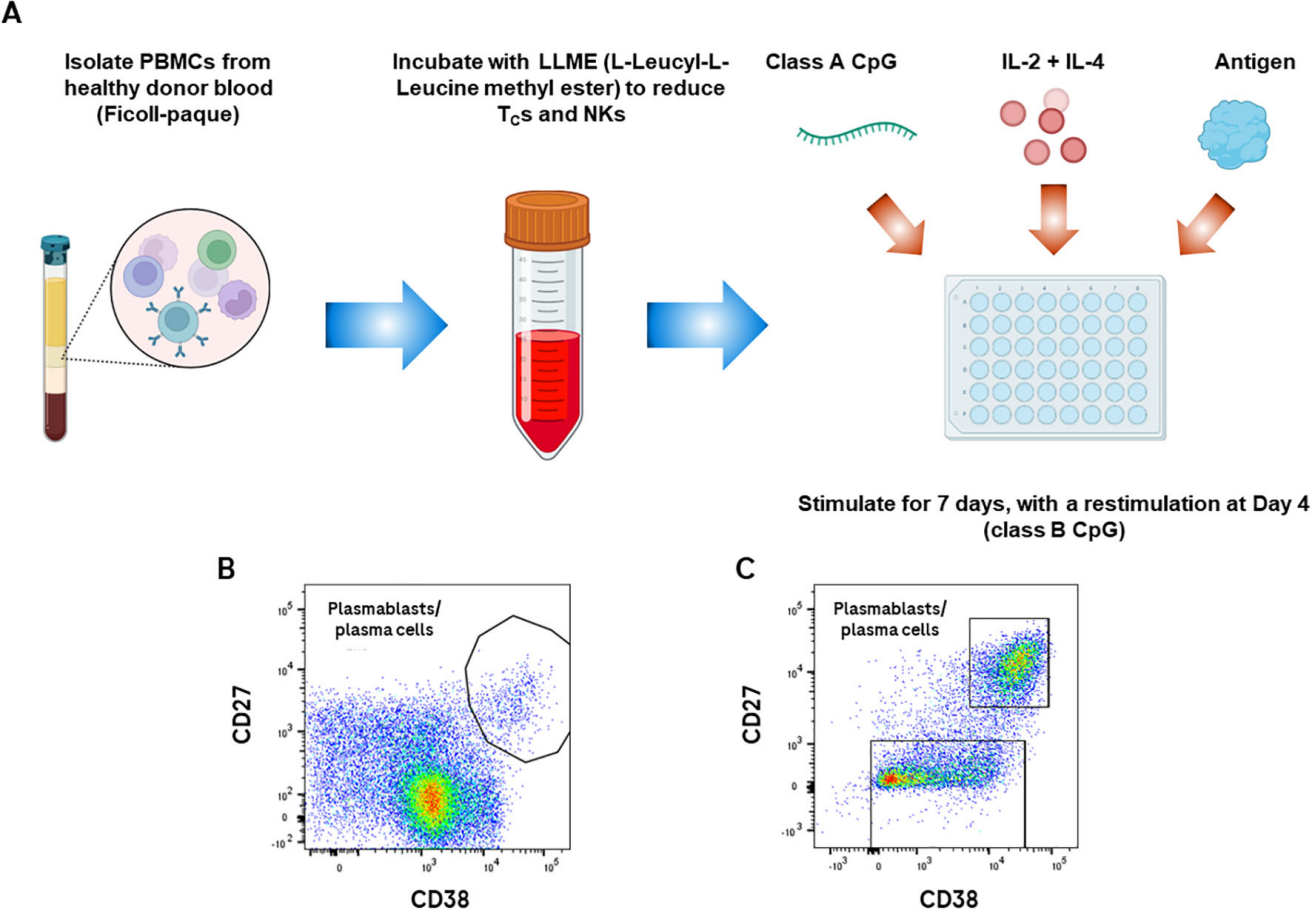
*Ex vivo* B-cell immunogenicity assay results in generation of plasmablasts/plasma cells after 7 days of stimulation. Schematic - PBMCs are isolated from healthy donors on a Ficoll gradient, then incubated with LLME to reduce TCs and NKs. Depleted PBMCs are then incubated with Class A CpG, IL-2, IL-4, and antigen. Cells are restimulated at Day 4 with Class B CpG, IL-2, and IL-4. Flow cytometric and/or confocal analysis is performed at Day 7 **(A)**. Representative plot of CD38 vs CD27 on healthy donor PBMCs before stimulation **(B)** vs after 7 days of stimulation with an immunogenic drug **(C)**. Samples were gated for lymphocytes on FSC/SSC, for single cells on SSC-A/SSC-H, CD45^+^ cells, then CD3^neg^CD19^+/lo^.

We developed a B-cell immunogenicity assay based on administration of known stimulators of B-cell development and antibody secretion – namely, IL-2, IL-4, and CpG – to PBMCs in culture, in the presence of benchmark drugs. The choice to use whole PBMCs is due to the need for accessory cells to support B-cell differentiation and survival via antigen presentation, co-stimulation, and cytokine secretion; the T-cell dependent antigen KLH was chosen as a positive control. A pre-incubation step with L-Leucyl-L-Leucine methyl ester (LLME) reduces the proportion and activity of cytotoxic T-cells and NK cells (15), leaving helper T-cells and monocytic lineage cells as the primary cells to interact with B-cells (**Figure 1A**).

This assay leads to the expansion of antibody-secreting cells (ASC) over the 7 days of culture, defined as CD45^+^CD3^neg^CD19^+/low^CD27^+^CD38^high^ lymphocytes (**Figures 1B, C**). These ASCs developed regardless of the presence or absence of externally administered antigen; this finding is likely due to endogenous antigens present in the blood of healthy donors. We therefore focused on tracking antigen-specific B-cells as the most relevant readout, via two methods. The first method relied on tracking the binding of antigen to BCR on antigen-specific cells, using an anti-antigen antibody to detect antigen bound to antigen-specific B-cells. The second method was to detect antigens either bound to specific B-cells or internalized by BCR, using antigen directly conjugated to a fluorophore. Both methods detected a subset of total ASCs that varied depending on the stimulation (**Table 1**).

**Table 1:** Median antibody-secreting cells (ASC) and Antigen^+^ cell percentages.

Stimulation	% ASC	% Antigen^+^ cells
No antigen	18.0	0.4
KLH	28.3	7.4
Adalimumab	7.3	10.9
Bevacizumab	6.1	9.0
CEA-IL2v	26.1	20.8
CEA-IgG	9.7	4.1
Roche product 1	19.3	9.3
Roche parental 1	18.6	2.8
Durvalumab	15.5	4.5
Infliximab	6.7	15.6
Natalizumab	7.8	14.2
Roche product 2	10.8	8.1
Roche parental 2	15.4	1.0

Clinical ADA rates were determined as previously described (16). For the Roche products that have not gone through the licensing process, the ADA rate used was what was seen in the single multi-dose clinical trial for each product (**Table 2**). The Roche parentals that were noted were antibodies with the same antigen binding as the product, but without a key structural moiety necessary for function.

**Table 2:** Clinical ADA rates.

Drug	Trade name	Format	Target	Clinical ADA rate
Adalimumab	Humira	Human	TNFα	23.0
Bevacizumab	Avastin	Humanized	VEGF	0.6
CEA-IL2v		Humanized	CEA	70.0
Roche product 1				100.0
Durvalumab	Imfinzi	Human	PD-L1	3.0
Infliximab	Remicade	Chimeric	TNFα	27.0
Natalizumab	Tysabri	Humanized	Integrin α4	6.0
Roche product 2				75.0

**Figure 2:**
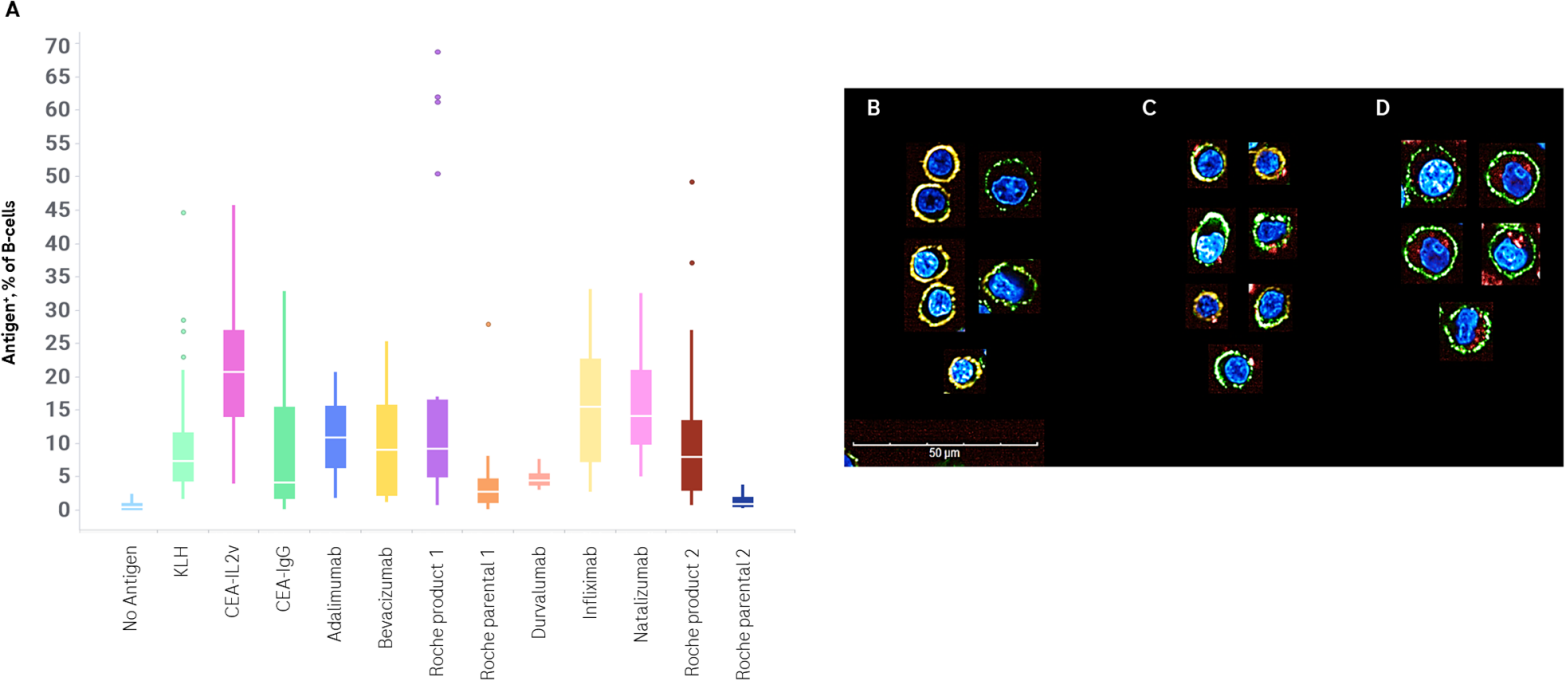
Flow cytometric and confocal analysis of drug-specific B-cells. PBMCs were stimulated with no exogenous antigen, KLH, or a set of drugs for 7 days. Drug specific B-cell frequency was determined by anti-PGLALA staining or incubation with fluorophore-conjugated drug, and replicate experiments plotted **(A)**. Confocal images of B-cells isolated from Day 7 ex vivo immunogenicity cultures after stimulation with no antigen **(B)** or CEA-IL2v **(C, D)**. Cells were stained with a nuclear Hoechst stain (blue), an anti-CD19 Alexa Fluor 488 (green), and an anti-CD79b PE (orange). Antigen was visualized either by an anti-PGLALA Alexa Fluor 647 **(C)** or direct conjugation of antigen to Alexa Fluor 647 **(D)** (red). Images are representative of cells acquired on an Opera Phenix using a 63× water immersion lens (maximum projection of 3 μm stack).

The percentage of Ag^+^ B-cells for all compounds tested is shown in **Figure 2A**. The most consistently high frequency of drug-specific B-cells was seen in cultures administered CEA-IL2v. This compound was chosen as a benchmark due to its high level of immunogenicity *in vivo*, with 70% of patients developing ADAs after dosing (17) (**Table 2**). Durvalumab was chosen as a benchmark on the low end of immunogenicity, with 3% of patients developing ADAs after dosing (**Table 2**).

In order to visualize antigen binding and uptake, B-cells were isolated after a 7-day culture. Antigen-specific B-cells were identified, as noted above, by two methods – staining with a labeled antigen-specific antibody, or by using directly labeled antigen. Cells stimulated in the absence of drug were used as a control. Cells were also stained with anti-CD19, anti-CD79b, and a nuclear stain, to facilitate identification. While many B-cells were phenotypically naïve/memory cells, being small, round, and with substantial surface staining of both CD19 and CD79b, ASCs – larger asymmetric CD19^+^CD79b^low^ cells – were also identified.

In the samples where antigen-specific cells were identified via a labeled anti-drug antibody, antigen labeling was confined to the cell surface. In the samples where antigen-specific cells were identified via labeled antigen, this labeled antigen was seen frequently in the cytoplasm of cells (**Figures 2B–D**).

### 3.2 CEA-IL2v does not promote immunogenicity in *trans*

**Figure 3:**
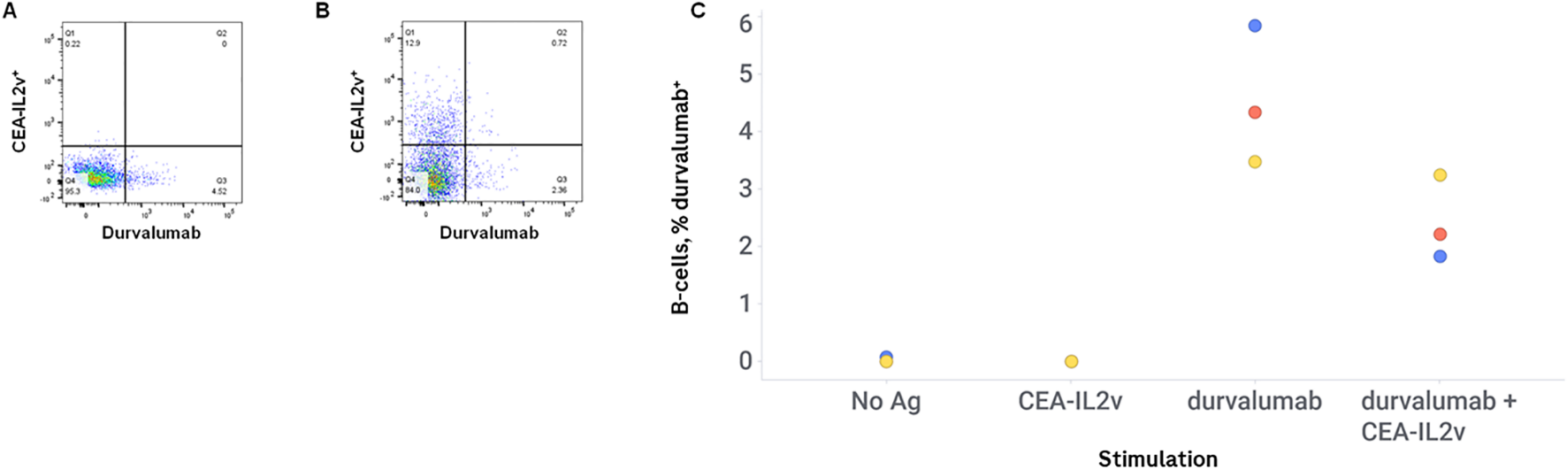
CEA-IL2v does not promote immunogenicity in *trans*. PMBCs were stimulated with durvalumab alone, or durvalumab + CEA-IL2v. Drugs were conjugated to different fluorophores (CEA-IL2v Alexa Fluor 647, durvalumab Alexa Fluor 680) to enable visualization of antigen-specific cells for the two drugs in the same well. Representative flow plots of durvalumab-stimulated cells **(A)** and CEA-IL2v + durvalumab-stimulated cells **(B)**, and percentages of durvalumab-positive B-cells in various stimulation conditions **(C)** are shown.

It is possible that the high response observed in this *ex vivo* B-cell assay to CEA-IL2v is due to immune system activation by the conjugated IL-2, and that therefore any immune activation seen in this *ex vivo* assay by CEA-IL2v would be as potent to co-cultured drugs as it is to CEA-IL2v itself. To determine if the robust B-cell response seen in this assay with CEA-IL2v is due to nonspecific activation of B-cells by its IL-2 moiety, PBMCs were stimulated with either CEA-IL2v or durvalumab (a drug with both low ADA incidence and low response in this *ex vivo* assay), or both in the same stimulation well. The low response to durvalumab was not increased by co-stimulation with CEA-IL2v, with the frequency of antigen-specific B-cells maintained at the same level or even reduced when co-incubated with CEA-IL2v, with antigen-specific percentages ranging from 3.5 to 5.8 in the absence of CEA-IL2v, and 1.8 to 3.2 in the presence of CEA-IL2v (**Figure 3**).

### 3.3 T-cell phenotype in B-cell immunogenicity assay

**Figure 4:**
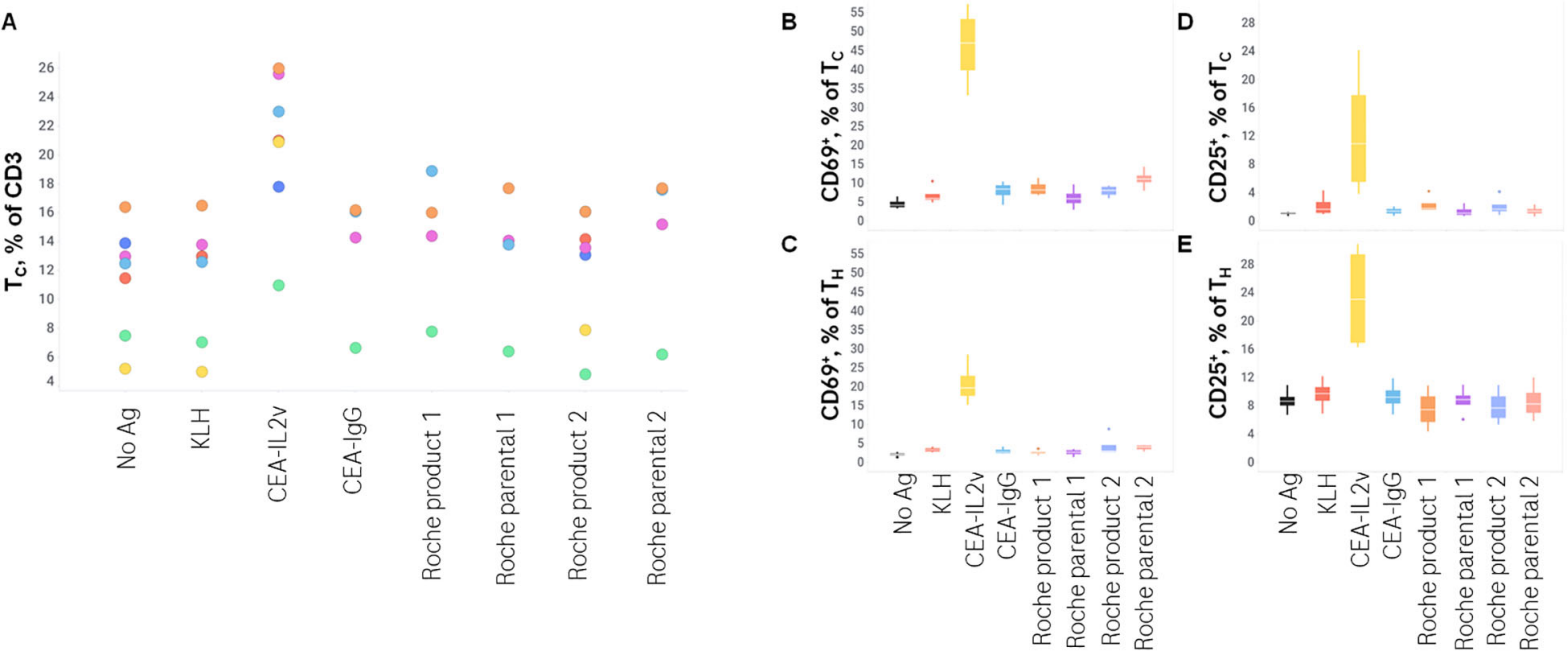
T-cell phenotype after stimulation. T_C_/T_H_ T-cell profile and CD25 and CD69 expression on these subsets was assessed by flow cytometry after 7 days of stimulation with various drugs. **(A)**, T_C_ as a % of total CD3^+^ lymphocytes; **(B)** CD69^+^ as a % of T_C_; **(C)** CD69^+^ as a % of T_H_; **(D)** CD25^+^ as a % of T_C_; **(E)** CD25^+^ as a % of T_H_.

As T-cell involvement is a key component of a humoral immune response, T-cells were phenotyped after 7 days of stimulation with a subset of drugs with the lowest and highest response of B-cells. CEA-IL2v, in agreement with published data in mice (18), resulted in a preferential expansion of cytotoxic T-cells (T_C_), with a median of 24% T_C_ per total T-cells versus 13% in the control. CEA-IL2v also resulted in a profound activation of both T_C_ and helper T-cells (T_H_), as revealed by a substantial upregulation of CD25 and CD69 expression compared to the controls (**Table 3**). The only other drug tested that increased T-cell activation by those cell surface markers was Roche Product 2, which increased CD25 on T_H_ and T_C_ (**Figure 4**).

**Table 3:** CD25 and CD69 expression on T-cell subsets.

Drug	Subset	Marker	Median % positive
No Antigen	T_C_	CD25	1.1
	T_C_	CD69	4.2
	T_H_	CD25	8.6
	T_H_	CD69	2.1
KLH	T_C_	CD25	1.7
	T_C_	CD69	5.9
	T_H_	CD25	9.7
	T_H_	CD69	3.3
CEA-IL2v	T_C_	CD25	10.9
	T_C_	CD69	47
	T_H_	CD25	23
	T_H_	CD69	19.7
CEA-IgG	T_C_	CD25	1.4
	T_C_	CD69	8.4
	T_H_	CD25	9.2
	T_H_	CD69	2.8
Roche product 1	T_C_	CD25	1.8
	T_C_	CD69	8.2
	T_H_	CD25	7.5
	T_H_	CD69	2.4
Roche parental 1	T_C_	CD25	1.1
	T_C_	CD69	5.8
	T_H_	CD25	8.9
	T_H_	CD69	2.7
Roche product 2	T_C_	CD25	1.6
	T_C_	CD69	8.1
	T_H_	CD25	7.7
	T_H_	CD69	3
Roche parental 2	T_C_	CD25	1.4
	T_C_	CD69	11.1
	T_H_	CD25	8.3
	T_H_	CD69	4

**Figure 5:**
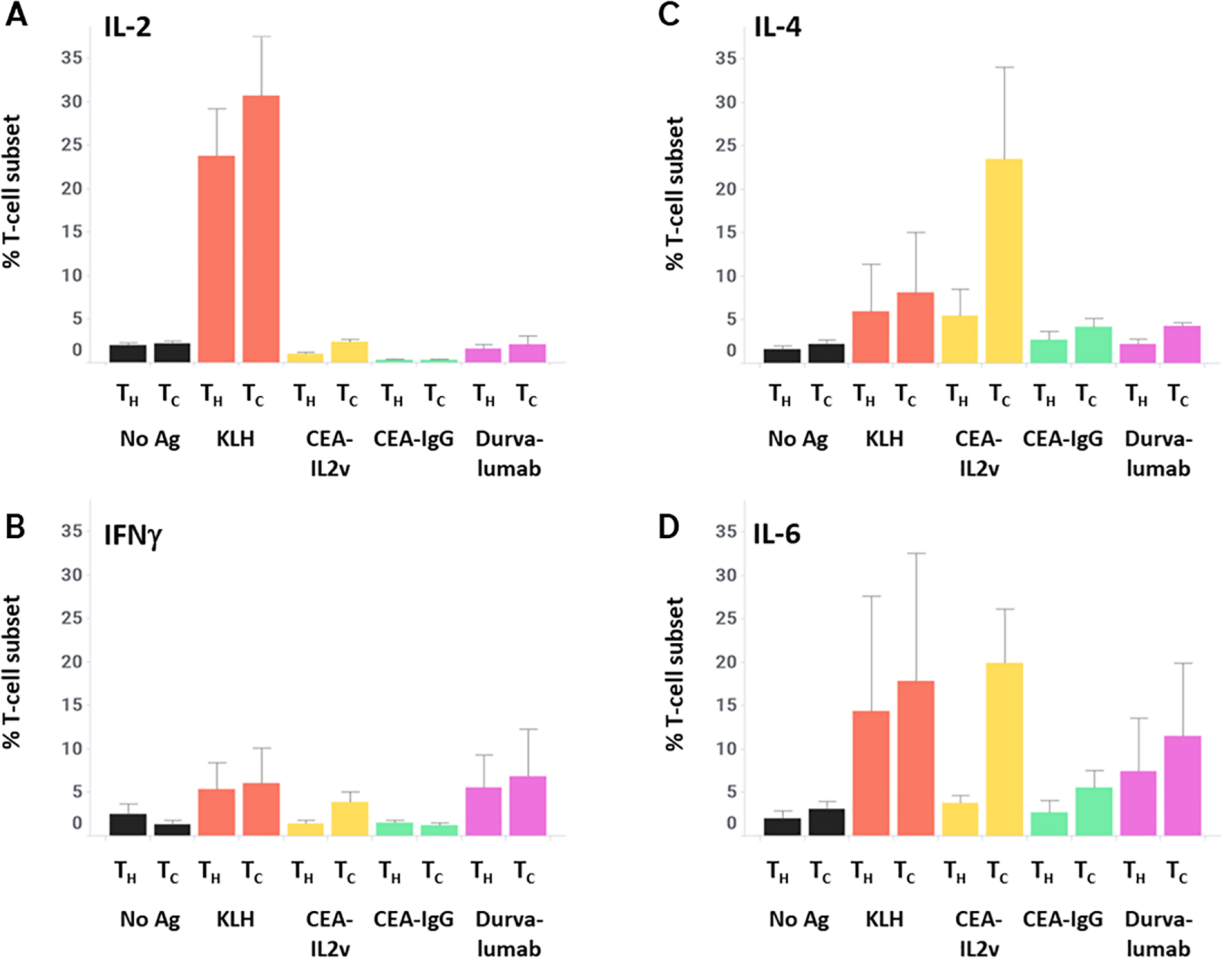
T-cell cytokine profiles after stimulation. After 7 days of stimulation with various drugs, PBMCs were incubated for 4 hours with Brefelden A, then stained intracellularly for the cytokines IL-2 **(A)**, IFNγ **(B)**, IL-4 **(C)**, and IL-6 **(D)**.

As these data suggest a differential activation of T-cells depending on antigen stimulation, the cytokine profile of T-cells was assessed after 7 days of culture followed by 4 hours of Brefeldin A (a protein transport inhibitor commonly used to amplify signals from intracellular cytokine staining by inhibiting transport mechanisms during cell activation), in order to characterize this activation in more detail. Intracellular cytokine staining revealed a low basal level of IL-2 expression (median 2% of T_H_ and T_C_), only increased by the KLH positive control (19% of T_H_ and 31% of T_C_), and a low basal level of IFNγ expression (2% of T_H_ and 1% of T_C_) that was not substantially increased by any stimulation assessed. In contrast, KLH had a minimal effect on IL-4 and IL-6 while CEA-IL2v increased those two cytokines in T_C_ specifically (2% to 31% for IL-4, 4% to 23% for IL-6) (**Figure 5**). These data demonstrate the utility of investigating the T-cell response beyond IFNγ secretion, as more humoral-related cytokines (IL-4, IL-6) were better-associated with both the *ex vivo* B-cell response and clinical immunogenicity.

### 3.4 Identification of specific peptides presented on antigen-specific B-cells

**Figure 6:**
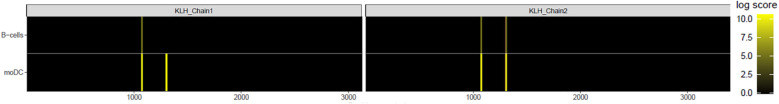
MAPPs of KLH^+^ B-cells demonstrate presentation of KLH-derived peptides. After 7 days of stimulation with KLH, antigen-specific B-cells and moDCs were isolated via FACS, lysed, MHC Class II-DR immunoprecipitated, and MHC Class II-DR -associated peptides identified via mass spectrometry. Peptides were mapped to KLH by PEAKS software.

To confirm that drug uptake was due to BCR-mediated uptake and processing, B-cells and moDCs were FACS-sorted from cultures stimulated with KLH or without exogenous antigen as a control. Cells were lysed, and MAPPs was performed. The presence of KLH-derived Class II-associated MHC peptides was confirmed in these sorted B-cells (**Figure 6**), indicating that these cells were functionally capable of processing antigen in our system and presenting antigen-derived peptides to autologous T-cells in their capacity as professional APCs.

### 3.5 Development of a higher-throughput whole blood immunogenicity assay

A higher-throughput whole blood-based assay was adapted from the PBMC-based assay; this assay used the same IL-2, IL-4, and CpG concentrations, and the same timing of stimulation, but omitted the LLME step to minimize processing steps and used 100 μL of whole blood in a 96-well plate. This assay yielded sufficient cells for antigen-specific B-cell enumeration via labeled drug uptake, although it did not yield enough for the downstream analyses that the PBMC-based assay allows.

**Figure 7:**
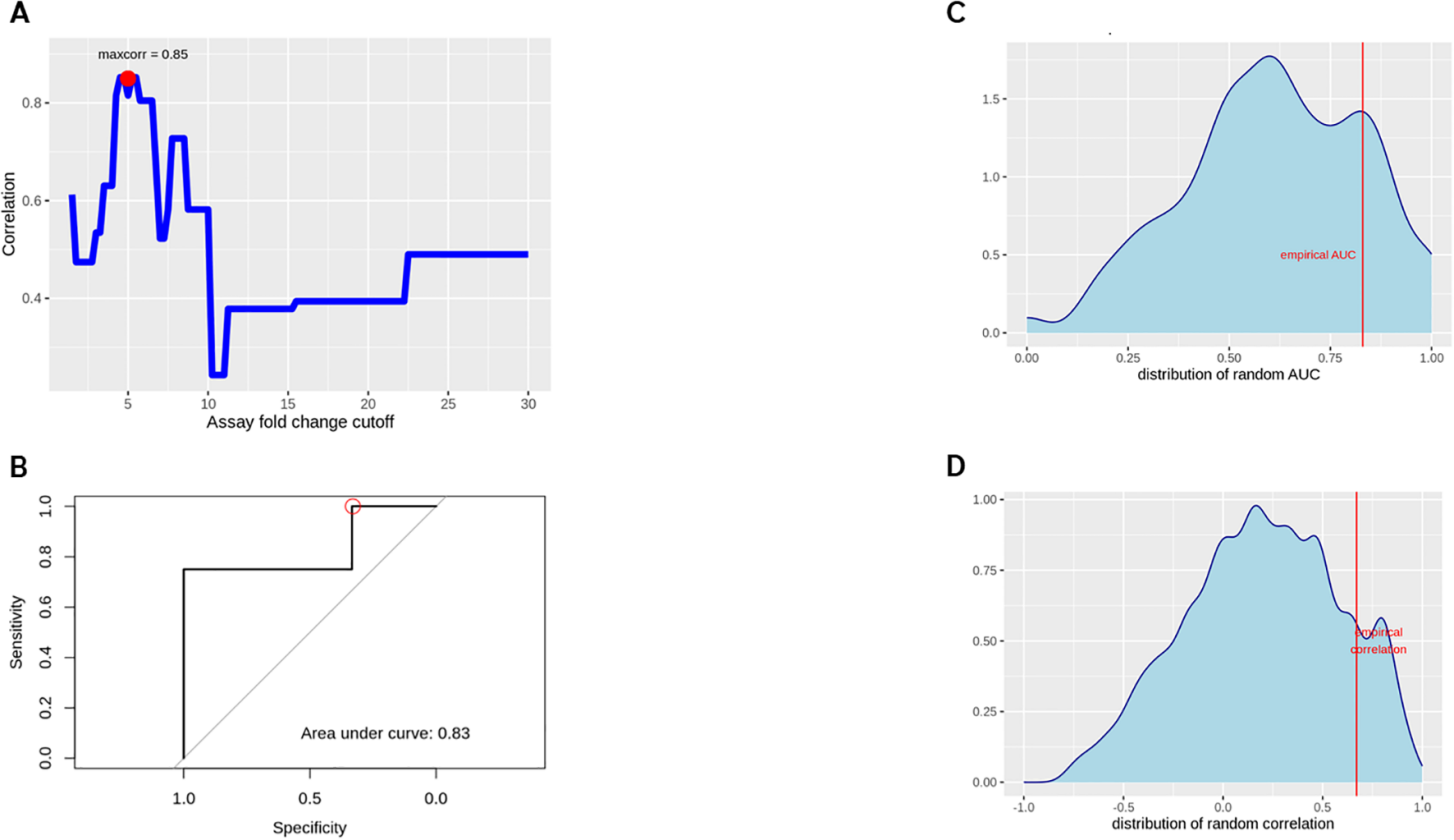
Spearman correlation between WBA and clinical response rates **(A)**, optimizing the correlation between assay and clinical response rates by varying the % positive B cell threshold to call a specific donor´s assay readout a positive response. A ROC curve for the empirically found ‘optimal setting’ (FC cutoff 5, ADA rate cutoff 15%) was generated **(B)**. A random permutation approach explored if any arbitrary assignment of clinical responses could be ‘predicted’ with comparable accuracy, for the empirical AUC **(C)** and response rate correlation **(D)**.

Preliminary assessments targeted to explore a potential predictive capability of this assay format yielded a maximum correlation for an assay fold change of 5.0, i.e. calling a donor with a corrected FC > 5 as positive (**Figures 7A, B**). This threshold leads to empirical donor positivity rates between 20% and 100% for the compounds tested.

Relating these numbers to a 15% clinical ADA threshold, we calculated a sensitivity of 100% and a specificity of 33% with regard to correctly identifying compounds with a risk of immunogenicity. Significant overfitting due to the specifically optimized FC cutoff is expected at this stage, while a more stringent cross validation is not feasible with the given number of compounds.

To assess nevertheless whether this approach actually yields a better-than-random result, the analysis was repeated 10.000 times with random permutations of the clinical ADA incidences. This led to a number of ‘best AUC’ and ´best correlation’ results (**Figures 7C, D**). This analysis reveals that in 21.47% of the runs we could achieve AUC values equal to or greater than the observed 0.83 when tuning the cutoffs for best apparent performance. Similarly, in 12.42% of the random trials we have an equal or better correlation coefficient between clinical and assay response rates than actually observed.

## 4 Discussion

Existing *ex vivo* immunogenicity assessments focus on peptides presented on dendritic cells (12) and the stimulation potential of these peptides to activate T-cells (10). Such assessments have greatly improved drug development, allowing molecules with a high immunogenicity potential to be de-prioritized or mitigated.

The B-cell response has been less well characterized. This response represents a critical axis to develop, as B-cells are the active source of ADAs. In addition to this role, B-cells themselves are professional APCs, capable of presenting drug-derived peptides to T-cells after internalizing drugs via BCR. Therefore, the development of assays that inform the B-cell mediated immunogenicity potential of therapeutics is a critical next step in the drug development process. The *ex vivo* assay described herein is conceptually straightforward, using a PBMC mixture treated with B-cell stimulating cytokines and CpGs. While straightforward, it demonstrated a number of different features of B-cell reactivity.

The first feature is generation of CD19^+^CD27^+^CD38^high^ B-cells, also shown on confocal imaging to have physical features of plasmablasts/plasma cells (PB/PC) and a loss of CD79b expression. This population preferentially expanded in our assay, becoming the dominant B-cell phenotype. While this population did expand in samples incubated without added antigen, it is important to note that this is likely in response to recently exposed/presented environmental antigens to which the healthy donors who provided the blood had been recently exposed. Interestingly, most donors assessed in the PBMC-based *ex vivo* assay (38/46) had low PB/PC in the control condition, while the remaining eight had PB/PC ranging from just over 30% to just over 60%, leading to a median of 18% (**Table 1**). It is therefore reasonable to hypothesize that most donors have a low baseline response, while a small subset with an active immune response (potentially due to vaccination, infection, or allergy) had a high baseline response. Future experiments will assess intra-individual variability to confirm this finding.

The second feature is expansion of B-cells specific for the drug. This feature was confirmed using two different methods of visualization. Anti-PGLALA was used to visualize BCR-bound drugs carrying a PGLALA mutation (19), which detected a quantifiable subset of B-cells; however, the persistence of cell-surface BCR could not be counted on to be consistent in this system. Firstly, BCR is often downregulated on activated B-cells exposed to antigen, as B-cells use BCR to internalize antigen. Secondly, B-cells maturing to PB/PC downregulate cell-surface BCR as they become primarily antibody-secreting cells. Therefore, we also confirmed uptake via incubation of stimulated cells with fluorescently labelled antigen.

While flow cytometry allows for quantification of these antigen-specific B-cells, it does not distinguish between cell-surface and internalized antigen. Therefore, confocal microscopy was employed to confirm that labelled antigen was taken up by the identified B-cells. This assessment demonstrated intracellular localization of labelled antigen, confirming uptake of labelled antigen by B-cells.

While uptake could be visualized by confocal imaging, and quantified via flow cytometry, the functional consequence of antigen uptake by BCR is the ability to process antigen and present it on MHC Class II receptors. We confirmed, via MAPPs, that this processing and presentation occurs in this *ex vivo* system. This is a particularly significant part of the process, as it is the means by which B-cells can orchestrate the immune response, enabling their own somatic hypermutation and affinity maturation, and eventually antigen spreading to encompass more of the therapeutic. While the MAPPs assay was used in our system to confirm the specificity of the differentiated B-cells, it can be used in future experiments to aid with the identification of immunogenic peptides, which either confirm (if the same peptides are presented by B-cells) or add to (if different peptides are presented by B-cells) the data generated from classic moDC-based MAPPs (11, 12).

This hypothesis-generating exercise will be repeated with more donors and compounds; however, it suggests that the whole blood assay leans heavily towards high sensitivity and lower specificity with regard to the clinical ADA rates, which is the optimal scenario for a hazard identification immunogenicity assay. We explored a number of different metrics, including % Ag+ B-cells, but found % Ag+ B-cells to be the most consistent metric and the one best correlated with high immunogenicity. This assay, given its prioritization of sensitivity over specificity, would work best as part of an overall ‘toolkit’ that includes in silico, MAPPs, DC-T, and peptide stimulation assays. Its utility is not limited to monoclonal antibodies; we have preliminary data that non-antibody proteins can also stimulate B-cells and be presented in this assay (data not shown).

The strongest response seen in our culture was to CEA-IL2v. While this has proven to be a highly immunogenic drug in clinic, with 70% of patients administered the drug developing ADAs (17) (**Table 2**), a possible explanation for the observation in our *ex vivo* assay is that the strong response seen to CEA-IL2v was nonspecific, due to liberation of the IL-2 moiety of the drug leading to a high concentration of free IL-2 in culture. To test this, CEA-IL2v was incubated with durvalumab, a drug with low immunogenicity in the clinic and weak stimulation in this *ex vivo* assay. If this response truly is a nonspecific consequence of liberated IL-2, we would expect to see the response to durvalumab to also be increased. However, compared to durvalumab stimulation alone, the response was, if anything, reduced, as the B-cells preferentially responded to CEA-IL2v. This supports that the immunogenicity to CEA-IL2v is directly due to the drug, rather than nonspecific to any drug incubated with additional IL-2.

A key feature of this assay is that the B-cells internalized, processed, and presented drug-derived peptides on MHC Class II. This feature allows peptides presented by B-cells to be identified and compared to those presented by moDCs, allowing B-cell specific liabilities that are not captured by traditional MAPPs to be identified. It also enables identification of liabilities that are potentially stronger for being shared across both cell types. The comparison of peptides presented on B-cells versus moDCs from the same donors is under investigation and is likely to substantially inform the drug development process. A key factor to consider when developing this assay for predictive use will be to include donors with an MHC background expected to cover the dominant MHCs expected in the drug-exposed population.

While the PBMC-based assay allows for the reported extensive downstream analyses of responding B-cells and supporting T-cells, a higher-throughput assay is more appropriate as a screening tool in early development. Therefore, we developed a whole blood-based assay that can be run with more donors and compounds in a 96-well plate format. In addition to being higher throughput, the cells are less manipulated and are in the presence of physiological serum factors, which may be the reason why the response in this assay was more robust and consistent. In this preliminary dataset, cutoffs for antigen^+^ B-cells and clinical ADA rates were generated, which can be tested in future experiments with more drugs with a variety of clinical immunogenicity rates.

Imperfect ADA prediction is to be expected for multiple reasons. The main reason is variability in clinical ADA rates, due to patient population, background medication, and even simply biological variability (16). Given that clinical ADA rates are highly variable, depending on these factors, a single assay can hardly capture the full spectrum of immunogenicity. Additionally, this assay was run on healthy donors. Donors without significant background disease or a history of disease treatment might be anticipated to have a more robust immune response than patients. However, this B cell-based immunogenicity assay is not intended to be a perfect predictor; it is intended to be one part of an integrated risk assessment strategy, and as such, a high sensitivity at the expense of a moderate amount of specificity is a useful feature.

Two critical aspects of drug development are the efficacy and the safety, which together inform the therapeutic window of the drug. Immunogenicity uniquely sits at the intersection of these two considerations, as immunogenicity has the potential to reduce exposure and pharmacodynamics, therefore efficacy, and can also mediate a harmful immune response, affecting safety. The immunogenicity toolbox has been substantially expanded in the last few years, but the B-cell side is still lacking robust hazard identification assays. The B-cell focused immunogenicity assay described in this paper adds this side of the immune system to the toolbox. Additional assay development is focused on moving this assay to a whole-blood format, allowing for a higher throughput assay to add to the preclinical risk assessment package. This work will potentially increase the efficacy of drugs that make it to the clinical trial phase, and increase the success rate of drugs overall.

## Data Availability

The datasets presented in this study can be found in online repositories. The names of the repository/repositories and accession number(s) can be found below: https://www.ebi.ac.uk/pride/archive/, PXD060948.
